# Seed Dormancy: Molecular Control of Its Induction and Alleviation

**DOI:** 10.3390/plants9101402

**Published:** 2020-10-21

**Authors:** Angel J. Matilla

**Affiliations:** Department of Functional Biology, Life Campus, Faculty of Pharmacy, University of Santiago de Compostela (USC), 15782 Santiago de Compostela, Spain; angeljesus.matilla@usc.es

**Keywords:** ROS, DOG1, physical dormancy, long-lived mRNA, monosomes, DNA methylation, auxin and ABA, alternating temperatures, GABA

## Abstract

A set of seed dormancy traits is included in this Special Issue. Thus, DELAY OF GERMINATION1 (DOG1) is reviewed in depth. Binding of DOG1 to Protein Phosphatase 2C ABSCISIC ACID (PP2C ABA) Hypersensitive Germination (AHG1) and heme are independent processes, but both are essential for DOG1’s function in vivo. AHG1 and DOG1 constitute a regulatory system for dormancy and germination. DOG1 affects the ABA INSENSITIVE5 (ABI5) expression level. Moreover, reactive oxygen species (ROS) homeostasis is linked with seed after-ripening (AR) process and the oxidation of a portion of seed long-lived (SLL) mRNAs seems to be related to dormancy release. The association of SLL mRNAs to monosomes is required for their transcriptional upregulation at the beginning of germination. Global DNA methylation levels remain stable during dormancy, decreasing when germination occurs. The remarkable intervention of auxin in the life of the seed is increasingly evident year after year. Here, its synergistic cooperation with ABA to promote the dormancy process is extensively reviewed. ABI3 participation in this process is critical. New data on the effect of alternating temperatures (ATs) on dormancy release are contained in this Special Issue. On the one hand, the transcriptome patterns stimulated at ATs comprised ethylene and ROS signaling and metabolism together with ABA degradation. On the other hand, a higher physical dormancy release was observed in *Medicago truncatula* under 35/15 °C than under 25/15 °C, and genome-wide association analysis identified 136 candidate genes related to secondary metabolite synthesis, hormone regulation, and modification of the cell wall. Finally, it is suggested that changes in endogenous γ-aminobutyric acid (GABA) may prevent chestnut germination, and a possible relation with H_2_O_2_ production is considered.

## 1. Introduction

The seed, a key entity in the life cycle of higher plants, allows and ensures its survival by acquiring primary dormancy (PD) during maturation [1]. The DELAY OF GERMINATION1 (DOG1) protein was identified and characterized as a major regulator of seed dormancy [2,3]. PD is the failure of seeds to germinate although environmental conditions are favorable. Interestingly, some PD-related genes are regulated through the epigenetic control of endosperm-specific gene expression [4,5]. Likewise, nondormant seeds can enter secondary dormancy (SD) upon exposure to unfavorable conditions for germination. Lack of light is a key factor involved in the induction of SD. However, it is not yet confirmed whether PD is a requirement to have the ability to acquire SD [6]. Recently, it was demonstrated that SD is induced in both high- and low-dormancy genotypes and that SD is less responsive to after-ripening (AR) and cold stratification than PD [7]. Maternal ABA is the only phytohormone known to induce, regulate, and maintain PD [8], and ABA levels and ABA signaling play pivotal roles in the regulation of PD and germination [9]. Furthermore, the ABA/ Gibberellins (GAs) balance is key to controlling PD and germination [10,11]. Thus, seeds of ABA-deficient mutants germinate faster than the wild-type ones, and transgenic plants constitutively expressing the ABA biosynthesis genes maintain deep PD [12]. Seed germination processes are under the control of classical phytohormones, reactive oxygen species (ROS) [13], brassinosteroids [14], strigolactones [15], as well as temperature, nitrate, and light [16,17]. Accordingly, PD and germination are strictly regulated by the modulation of suitable phytohormones, transcription factors, and environmental signaling networks [9]. This regulation mechanism is supposed to be highly conserved [18]. Together, PD and germination are two closely linked physiological traits that have great impacts on the adaptation and survival of seed plants. Although the phytohormones involved in these two traits have been largely identified, their mechanisms of interaction with external factors and how dormancy is broken under different conditions are more elusive. In this Special Issue, some aspects of the regulation of seed dormancy and germination are addressed.

## 2. Seed Dormancy and Delay of Germination-1 (DOG1) Protein

Due to the great repercussion of seed dormancy in the life of the seed, a great deal of research on PD has been developed in the last few decades. One of the reasons, among several others, is the appearance of pre-harvest sprouting (viviparism) in the mother plant when PD is not triggered. Viviparism is an important problem in cereal production because it reduces crop yield and quality. In other words, knowledge of the initiation, maintenance, and loss of PD is key to understanding how the germination process is triggered. The transcriptional and epigenetic control of dormancy, as well as the great advances in proteomics, have clarified a considerable number of PD mechanisms, which are essential to the survival of higher plants. In 2006, DOG1 was identified as a major Quantitative Trait Loci (QTL) for seed dormancy variability among natural *Arabidopsis thaliana* accessions, and *dog1* T-DNA insertional mutants exhibit reduced seed dormancy [19]. The expression of DOG1 is widely regulated and increases during seed maturation. DOG1 protein levels accumulate during the last phase of embryogenesis and correlate with the depth of PD. However, although DOG1 is relatively stable, DOG1 mRNA disappears quickly after seed imbibition. Given its key role in PD, DOG1 has been extensively studied in recent years. Currently, little is known about the precise molecular mechanism underlying the transcriptional regulation of *DOG1.* Carrillo-Barral et al. [20] present here a detailed update on DOG1. Their review focuses on why DOG1 is a key signaling molecule to coordinate seed life and, very specifically, the acquisition and loss of PD. DOG1 enhances ABA signaling through its binding to PP2C ABA Hypersensitive Germination (AHG1). Likewise, DOG1 suppresses the AHG1 action to enhance ABA sensitivity and impose PD. To carry out this suppression, the formation of the DOG1–heme complex is essential. In contrast, *dog1* mutant seeds, which have scarce endogenous ABA and high GA content, exhibit a non-dormancy phenotype. At the physiological level, DOG1 is tightly regulated by a complex array of transformations that include alternative splicing and polyadenylation, histone modifications, and a *cis*-acting antisense non-coding transcript (asDOG1). The activation of *DOG1* expression leading to increased PD requires that bZIP-transcription factor 67 (bZIP67) be bound to the DOG1 promoter. Although *DOG1* is mainly expressed in seeds, other organs are also capable of doing so.

## 3. ROS and Nucleic Acid Modifications during Seed Dormancy

Plants have to deal with ROS constantly generated in the cell organelles. Except for certain phases of the plant life cycle (e.g., dry viable seeds), the production of ROS is essentially associated with photosynthesis. When an excess of ROS is produced and a threshold exceeded (e.g., under stress conditions), cellular damage may arise and trigger cell death. To a greater or lesser degree, all ROS are markedly reactive. Thus, they are able to oxidize biological molecules, including lipids, DNA, RNAs, and proteins, RNA being more susceptible to oxidative damage than DNA. Interestingly, we now know that ROS is not always detrimental to the cell. This is what sometimes happens, for example, with the singlet oxygen (^1^O_2_). That is, the ^1^O_2_ generated in the light-harvesting complex (LHC) of the chloroplast grana core under excessive light energy, or in the photosystem II reaction center (PSII-RC) of the grana margins under low light energy, may act as a highly versatile signal (i.e., chloroplast-to-nucleus retrograde signaling (ChNRS)) triggering beneficial cell responses. To sustain life, an organism must maintain ROS homeostatic levels. This control involves more than 150 genes in *Arabidopsis*. In contrast, ROS has been correlated with a low degree of seed dormancy. When the ROS level reaches a certain threshold, dormancy is alleviated and the subsequent germination can be initiated. Likewise, seed aging takes place, and an intensive degradation of nucleic acids and proteins occurs. Interestingly, it was recently demonstrated that AtPER1, a seed-specific peroxiredoxin, eliminates ROS to suppress ABA catabolism and GA biosynthesis, and thus improves the PD and make the seeds less sensitive to adverse environmental conditions [21]. In this Special Issue, Katsuya-Gaviria et al. [22] review in detail the biological significance of nucleic acid oxidation caused by ROS during PD and germination. This update also refers to the state of the art regarding DNA and RNA methylation in seed biology. Thus, we can see how ROS increases upon after-ripening (AR) and dormancy release. Interestingly, ROS is located close to the radicle apex during imbibition, whereas oxidative species does not have a certain distribution in these dormant seeds. In support of this, several enzymes that participate in ROS homeostasis have been associated with the germination and AR process. ROS oxidizes nucleic acids at different molecular positions affecting their stability. It is known that 8-hydroxyguanosine (8-OHG) is the most habitual oxidative nucleoside in RNA molecules. Likewise, the oxidation of a fraction of seed long-lived (SLL) mRNAs seems to be related to dormancy release and seed aging. However, how the SSL mRNAs involved in germination are preserved from oxidation during dry seed storage is not yet clear. Recently, it was proven that ∼17% of the SLL mRNAs are specifically associated with monosomes and are translationally upregulated during seed germination. For this, the formation of the SSL mRNA–RNA binding protein (RBP)–monosome complex seems to be key in the process of safeguarding these SSL mRNAs. Together, the association with monosomes likely protects the SSL mRNAs needed during early seed imbibition in a state ready for translation. Their review clearly specifies all of the above. Furthermore, DNA methylation is a well-known epigenetic mechanism of controlling gene expression. During *A. thaliana* embryogenesis, there is a global increase in CHH-context methylation. Global DNA methylation levels remain stable during seed dormancy, decreasing when germination occurs. Up to now, the presence of specific DNA methylation markers associated with dormancy or germination transcriptomes remains to be elucidated.

## 4. Auxins and Seed Dormancy

In addition to higher plants, auxin is a signaling molecule that is present in living organisms such as algae, moss, liverworts, lycophytes, and microorganisms. Auxin is involved in multi-functional processes during plant growth and development. Recently, auxin signaling was thoroughly reviewed [23]. Regarding the seed, it is now widely accepted that auxin biosynthesis is required for an array of seed developmental processes (e.g., embryogenesis and endosperm development, among others). Current studies have elucidated that auxin is also involved in the transition from PD to germination. Recent studies have shown that auxin possesses positive effects on seed dormancy, being (in conjunction with ABA) the second known hormone that induces seed dormancy. Thus, it was demonstrated, for the first time at the molecular level, a role for auxin in PD through stimulation of ABA signaling, identifying auxin as a dormancy promotor [24]. Matilla (2020) [25] carries out here an in-depth review of the participation of auxin in embryogenesis, PD, and germination. The dynamic of expression and localization (i.e., proembryo, hypophysis, and suspensor) of several key genes for the biosynthesis and transport of auxins in the globular phase was carefully checked (Figure 1 of the review). The bHLH49 transcription factor appears to be a notable mediator of the auxin-dependent suppression of embryo identity in suspensor cells. Likewise, synthesis, transport, and compartmentalization of auxins are crucial for the ovule, endosperm, and seed-coat (SC) development. Auxin transport from the endosperm to the integuments is regulated by AGAMOUS-LIKE 62 (AGL62), the encoding gene of which is specifically expressed in the endosperm to suppress its cellularization. In the absence of AGL62 (i.e., agl62 mutants), auxin remains trapped in the endosperm and the SC fails to develop (i.e., seed abortion). The application of auxin represses soybean seed germination through decreasing the ABA/GA ratio. Jointly, it is suggested that auxin acts synergistically with ABA to promote PD and inhibit germination. Recent biochemical and genetic evidence supports the involvement of auxins in PD. In this process, the participation of the transcriptional regulator ABA INSENSITIVE3 (ABI3) is critical, revealing a cross-talk between auxin and ABA signaling. Recent information demonstrates that auxin acts downstream of ABA to promote seed germination. However, it is still unknown if any process exists in which ABA acts downstream of auxin. An exhaustive analysis of the auxin responsiveness of ABA biosynthesis, transport, and signaling mutants will be required for this.

## 5. Gene Expression Patterns and Physiological Response Associated with Release of Dormancy under Alternating Temperatures

Although alternating temperatures (ATs) are more effective than constant ones in stimulating germination of some seeds, little is known at the physiological and molecular level about the regulation of the breaking of dormancy by ATs. It now seems clear that the convergence between ROS signaling and classical phytohormones participates in this dormancy-breaking process. Huarte et al. [26] now turn their attention to this breaking mechanism using after-ripening cardoon seeds as a biological system. Previously, it was proven that fluctuating temperatures terminate dormancy in this seed by turning off ABA synthesis and reducing ABA signaling, but not stimulating GA synthesis or signaling [27]. In this work, an advance in the break dormancy knowledge is carried out through large-scale gene expression. The transcriptome patterns stimulated at ATs comprised ethylene and ROS signaling and metabolism together with ABA degradation. In parallel, the upregulation of ethylene metabolism under AT conditions is also supported by physiological analysis. Interestingly, ROS depletion hampers the breakage process.

## 6. Effects of GABA on the Germination of Recalcitrant Seeds: Implications on Primary Dormancy

γ-Aminobutyric acid (GABA), a non-protein amino acid, is an important component of the free amino acid pool of living organisms. The enzymes involved in its metabolism are evolutionarily very conserved. Recently, the GABA implications in plant growth and development have been updated [28]. Thus, genetic and physiological studies have proven that GABA is involved in barley aleurone metabolism. Furthermore, the scant current evidence on the mechanism by which GABA acts as a signaling molecule in plants has been also reviewed [29]. The recent identification of a GABA receptor indicates that GABA is a signaling molecule and not just a metabolite [30]. Vigabatrin is a specific GABA transaminase inhibitor that inhibits GABA degradation. In this Special Issue, Du et al. [31] now report that high GABA levels exist in the chestnut recalcitrant viable seeds before germination. Likewise, they also suggest that endogenous GABA may play a specific role in the germination. Exogenous GABA and vigabatrin induced an accumulation of H_2_O_2_, possibly contributing to the inhibition of chestnut seed germination. In parallel, the authors point out that this inhibition may be due to an alteration in the balance between carbon and nitrogen metabolism, especially the free amino acid contents before germination. Together, the results presented here suggest that changes in GABA levels in chestnut seeds may prevent seed germination.

## 7. Physical Dormancy Release in *Medicago truncatula* Seeds is Related to Environmental Variations

Physical dormancy is caused by water-impermeable palisade cells in the SC. It is frequent in legumes, and the factors that release this type of dormancy are hardly known [32]. Temperature and soil moisture oscillations are the major players under natural conditions. Renzi et al. [33] present a study on temperature-related physical dormancy release in seeds of *Medicago truncatula.* These seeds exhibit both physical and physiological dormancy, the latter being non-deep. Seed dormancy release varied among accessions and years, and this could potentially act as a mechanism that favors the persistence of the seed in the soil and helps to distribute genetic diversity through time. However, comparing the results obtained with others recently published, the authors suggest that dormancy is an adaptation securing population survival in less predictable conditions. Moreover, unpredictable natural environments can select earlier within-season germination phenology. On the contrary, although dormancy is genetically determined, it also depends on the environmental conditions experienced by the mother plant and the subsequent status of the seed. In this work, the authors carry out a detailed and in-depth discussion based on the results obtained and those previously published on the genetic basis of the release of seed dormancy in legumes.
